# Supplementary material for: FrangiPANe, a tool for creating a panreference using left behind reads
Source: NAR Genom Bioinform. 2023 Feb 20;5(1):lqad013. doi: 10.1093/nargab/lqad013 (PMC9940456; doi:10.1093/nargab/lqad013)
Supplement: lqad013_Supplemental_File [file lqad013_supplemental_file.pdf]

SUPPLEMENTARY DATA

Supplementary Notes

Evaluation of ABYSS Assembly using variable k-mer size. We performed assembly of pair-ended short reads from the accession AA using abyss-pe with k-mer size (option -k) ranging from 20 to 64 bp (Supplementary Table 1). The contigs number and the L50 were the lowest and the N50 was the highest for a k-mer size of 64 bp.

Optimization of parameters to cluster contigs using CD-HIT. We optimized the following two CD-HIT parameters: percent identity cutoff (option -c) and length difference cutoff (option -s) using gene sequences from 8 *O. barthii* accessions. For each accession, the polymorphism in each gene was identified using the same approach described in Cubry et al. (2018). Polymorphisms were filtered with GATK SelectVariants (options -env -trimAlternates). We extracted all the gene sequences using GATK fastaAlternateReferenceMaker, which represented a total of 302,240 sequences (8 sequences for each gene out of 37,788 annotated).

Next, CD-HIT was used to cluster gene alleles, with values of -c and -s parameters ranging from 0.8 to 0.95 (Supplementary Table 2). We choose a value of -c and -s at 0.80 and 0.95 which produced 37,715 clusters, the closest value to the expected number of genes.

Supplementary Tables

k-mer length	#contigs	L50	N50	Max contig length	#bp
20	181,263	19,456	90	6,898	11,034,892
25	125,182	10,043	149	14,379	11,003,011
30	92,010	6,089	233	17,863	10,761,235
35	70,530	3,981	342	23,451	10,522,680
40	56,249	2,791	463	21,510	10,284,808
45	50,086	2,482	516	29,056	10,526,003
50	40,370	1,816	680	29,056	10,081,504
55	34,491	1,495	812	40,740	9,786,312
60	29,532	1,263	934	29,194	9,414,536
64	25,350	1,059	1,279	38,469	8,994,500

Supplementary Table 1. Assembly of AA reads performed with different k-mer length. Different metrics were calculated : contigs number (#contigs), L50 (number of the biggest contigs required to cover 50% of the total assembly length), N50 (the sequence length of the shortest contig required to cover 50% of the total assembly length), maximum contig length, total size in bp (#bp)

	-s 0.8	-s 0.85	-s 0.9	-s 0.95
**-c 0.8**	37,097	37,250	37,408	37,715
**-c 0.85**	38,100	38,220	38,342	38,574
**-c 0.9**	39,246	39,329	39,413	39,579
**-c 0.95**	40,876	40,930	40,977	41,053

Supplementary Table 2. Number of clusters produced by CD-HIT with the options -c (percent identity cutoff) and -s (length difference cutoff) values ranging from 0.8 to 0.95

Software Name	Version	url ref
Docker		https://docs.docker.com/get-docker/
Jupyter<br>Python<br>biopython	3.9.7	http:/www.python.org<br>https://biopython.org/
ea-utils	1.01	https://expressionanalysis.github.io/ea-utils/
bwa	0.7.17	https://github.com/lh3/bwa/blob/master/README.md (34)
samtools	1.10	http://www.htslib.org/ (35)
abyss	2.0	https://github.com/bcgsc/abyss/blob/master/README.md (36)
assembly-stats	1.01	https://github.com/sanger-pathogens/assembly-stats
CD-HIT	4.8.1	https://github.com/weizhongli/cdhit/blob/master/README (38)

Supplementary Table 3. List of main tools required by frangiPANe.The complete list with python packages is available at https://github.com/tranchant/frangiPANe

	interspecies	*O. barthii*	*O. glaberrima*
**#total contigs**	484,394	299,408	184;986
**#singleton**	273,126	170,980	102,146
**#cluster**	211,268	128,428	82,840

Supplementary Table 4. Number of non redundant contigs (singleton and clustered sequences) given by CD-HIT using 80% and 95% as thresholds for sequence identity and coverage respectively.

	Panreference	CG14 genome	Contigs
**% TE**	52.1	29.2	67.5
**% Retrotransposons**	25.3	18	30.1
**% DNA transposons**	16.3	7.7	22.2
**% Unclassified elements**	10.5	3.5	15.2

Supplementary Table 5. The ratio of total transposable element content (%TE) of the panrenference, the CG14 genome and the non redundant contigs. The ratio of major classes of transposable elements (Retrotransposons, DNA transposons, unclassified elements) are also provided.

	mean	median
**Sequence identity**	0.96	0.99
**Alignment coverage**	0.98	1.0

Supplementary Table 6. Mapping of genes from the Nipponbare genome on the panreference with the software liftoff. The table shows the mean and median for sequence identity ratio and alignment coverage in exons, respectively.

GO ID	GO Name	FDR	GO Category R	P-Value
GO:0051674	localization of cell	0.95	BIOLOGICAL PROCESS	0.04531
GO:0048870	cell motility	0.95	BIOLOGICAL PROCESS	0.04531
GO:0040011	locomotion	0.95	BIOLOGICAL PROCESS	0.04531
GO:0043436	oxoacid metabolic process	0.59	BIOLOGICAL PROCESS	0.01979
GO:0019752	carboxylic acid metabolic process	0.59	BIOLOGICAL PROCESS	0.01979
GO:0006520	cellular amino acid metabolic process	0.59	BIOLOGICAL PROCESS	0.01979
GO:0006082	organic acid metabolic process	0.59	BIOLOGICAL PROCESS	0.01979
GO:0098754	detoxification	0.13	BIOLOGICAL PROCESS	0.00185
GO:0042221	response to chemical	0.13	BIOLOGICAL PROCESS	0.00185
GO:0009636	response to toxic substance	0.13	BIOLOGICAL PROCESS	0.00185

Supplementary Table 7. List of the significantly enriched gene ontology terms among genes present in the contigs compared to all panreference genes.

Supplementary Figures

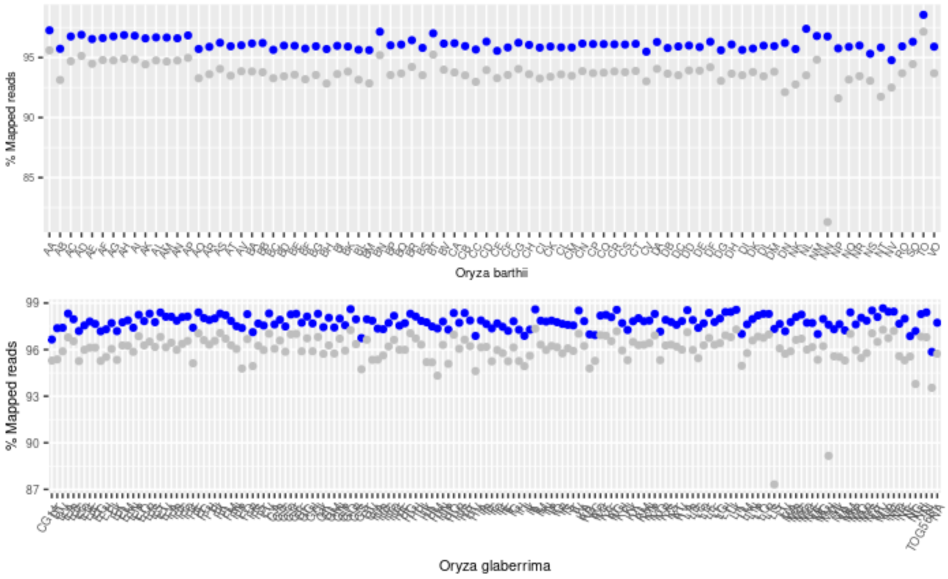

Supplementary Figure 1. Mapping statistics of 248 african rice (164 domesticated and 84 wild relatives). Each sample is plotted with its respective percentage of mapped reads (blue) and reads correctly mapped in pair (grey) for *Oryza barthii* (top) and *O. glaberrima* (bottom) respectively.

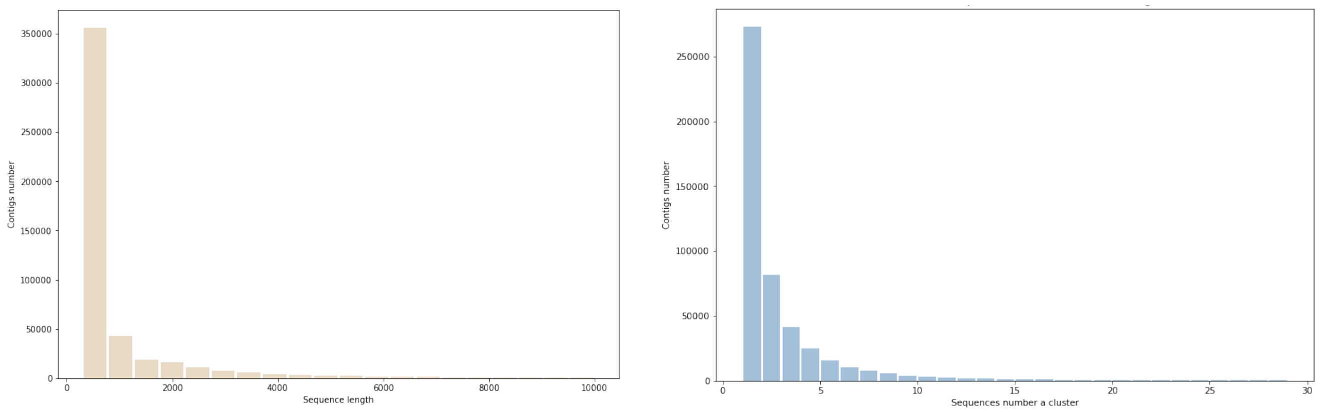

(a) (b)

Supplementary Figure 2. Reducing redundancy.

(a) Distribution of the sequence length (bp) of the contigs (singleton and cluster) after removing redundancy with cd-hit (with a maximum length set at 10,000 pb). The average sequence size was 1,060 bp.

(b) Distribution of the singleton number and and the sequence number per cluster

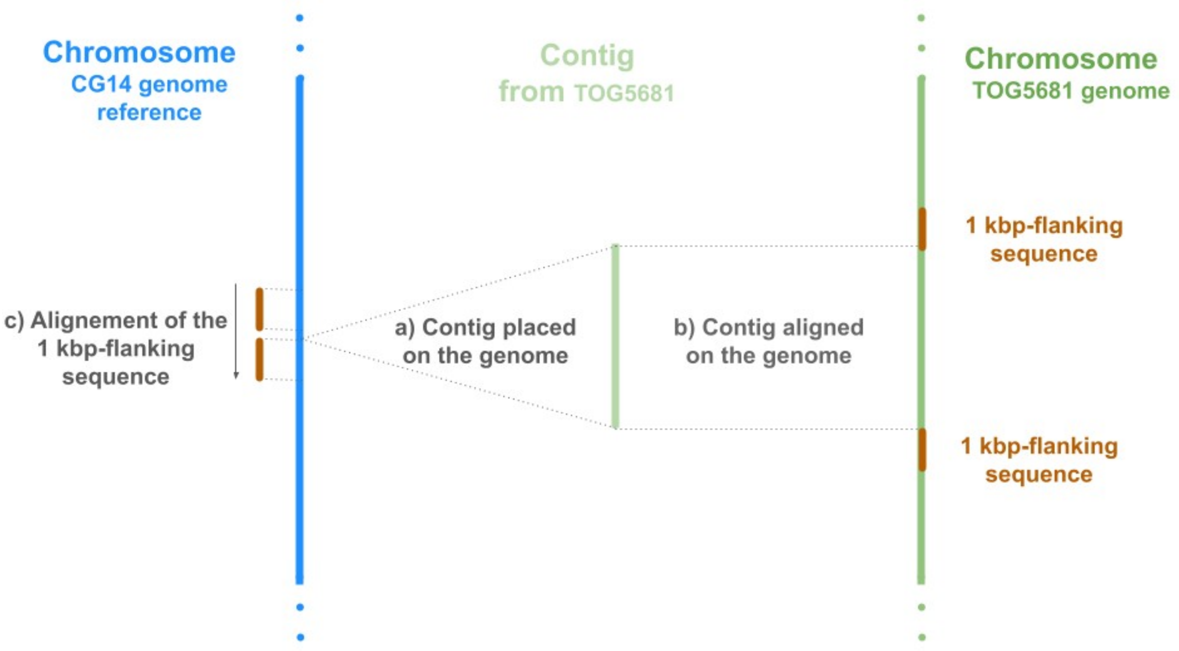

Supplementary Figure 3. Validation of the anchoring approach.

The contigs from TOG5681 obtained by assembling short reads unmapped to the CG14 genome reference (a) were aligned on a ONT assembly of TOG5681 genome, (b) the TOG5681 1kbp-flanking sequences surrounding the aligned contigs were remapped against the CG14 genome reference to check if they were found at the same position on the CG14 genome in a 1 kbp-window of the position where the contig was anchored (c).

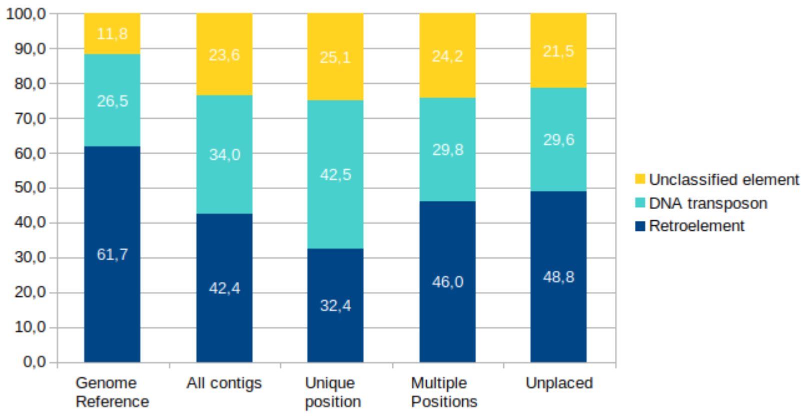

Supplementary Figure 4. Relative proportions of repeat elements

Considering only repetitive elements, this plot shows the relative proportions of transposable element classes (Retroelement, DNA transposon and unclassified repetitive element) on the reference genome, all the contigs and only the contigs that were placed on the reference genome (single or multiple positions) or unplaced.

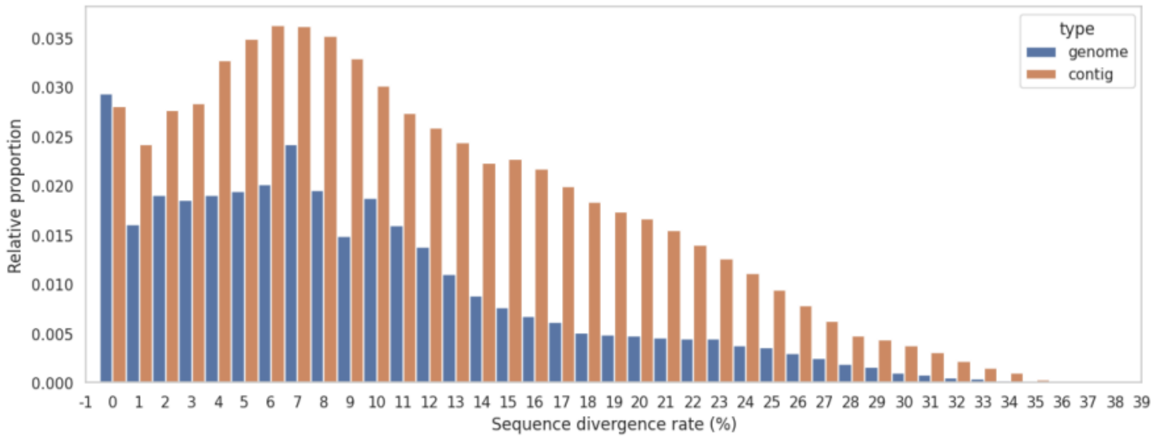

Supplementary Figure 5. Sequences divergence rate of repeats in the genome and the contigs.

The x-axis represents the sequence divergence rate of repeats (DNA transposons and Retrotransposons) annotated by RepeatMasker. The y-axis represents the relative proportion of the different divergent classes in the genome and in the contig.

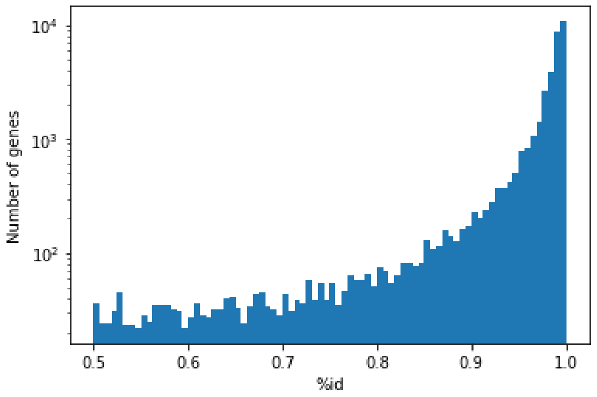

(a)

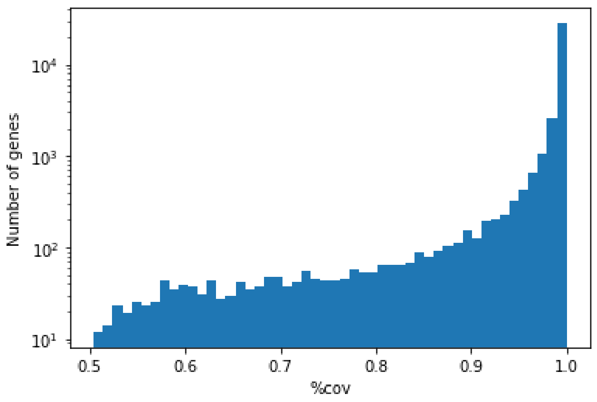

(b)

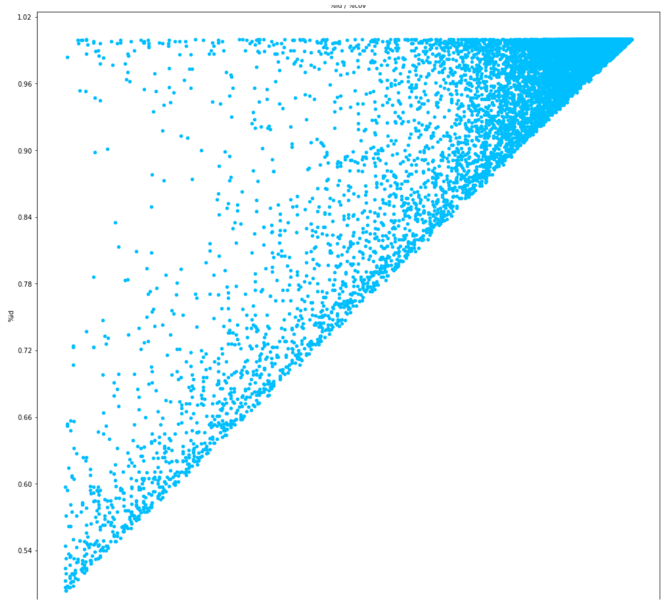

(c)

Supplementary Figure 6. Genes mapping.

(a) Distribution of the Nipponbare and the panreference sequence identity. This histogram shows the distribution of exon sequence identity of genes.

(b) Distribution of the Nipponbare and the panreference alignment coverage in exon. This histogram shows the distribution of alignment coverage in exons.

(c) Plot of the sequence identity sequence identity ratio and alignment coverage in exons between the Nipponbare genome and the panreference.

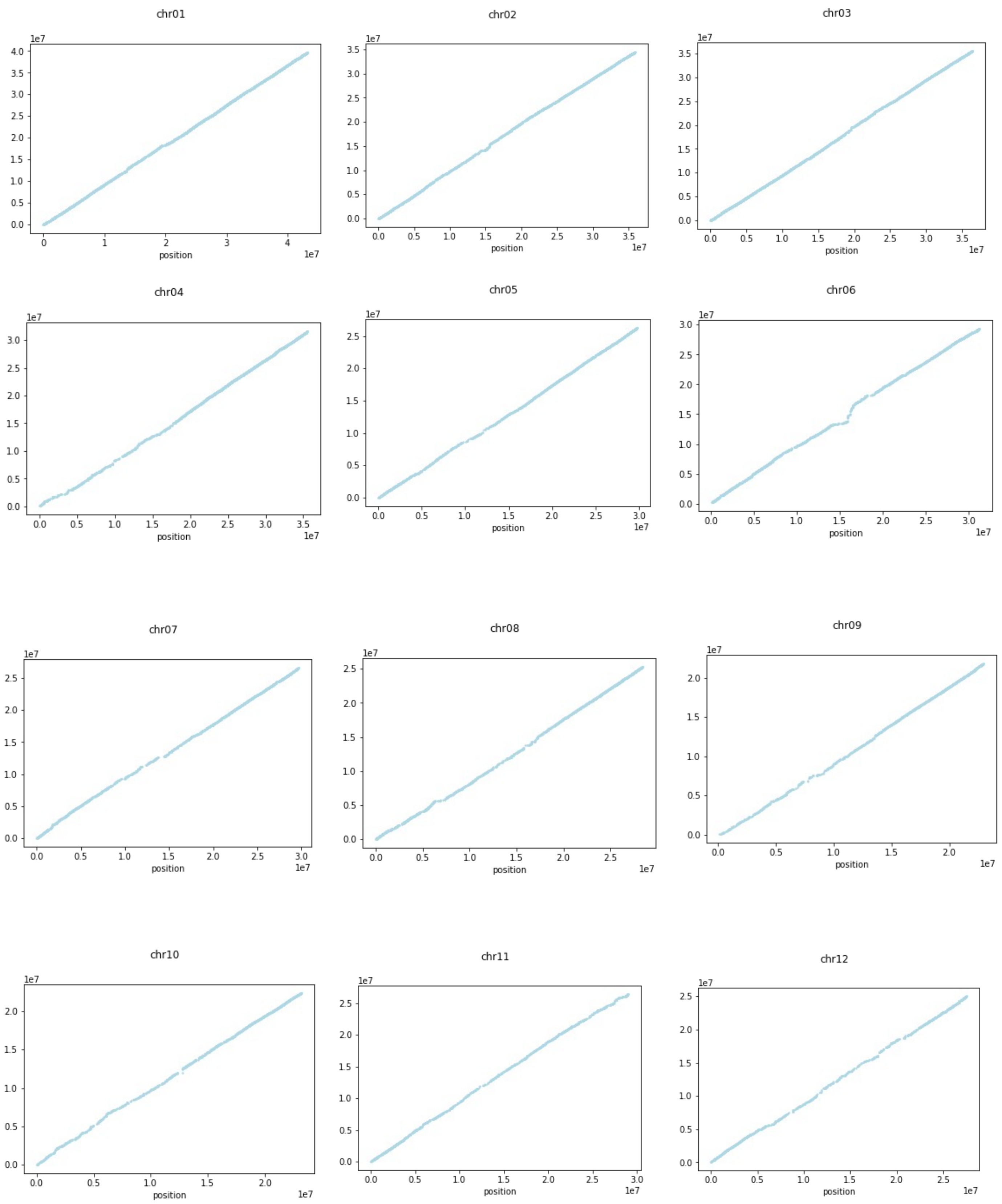

Supplementary Figure 7. The Nipponbare and the CG14 gene order. Each dot plot shows the position of each gene on the CG14 (x-axis) and the Nipponbare (y-axis) chromosome.
